# Supplementary material for: In Vitro Anti-Influenza A Virus H1N1 Effect of Sesquiterpene-Rich Extracts of Carpesium abrotanoides
Source: Molecules. 2022 Nov 29;27(23):8313. doi: 10.3390/molecules27238313 (PMC9739900; doi:10.3390/molecules27238313)

Supplementary Materials

In Vitro Anti-Influenza A Virus H1N1 Effect of Sesquiterpene-Rich Extracts of *Carpesium abrotanoides*

Li Li [1,†], Shenghui Yang [2,†], Dilu Chen [1], Zhihuang Wu [2], Meijun Zhang [2], Fang Yang [3], Li Qin [1,4,*] and Xiaojiang Zhou [1,*]

1 College of Pharmacy, Hunan University of Chinese Medicine, Changsha 410208, China
2 College of Medicine, Hunan University of Chinese Medicine, Changsha 410208, China
3 Changde Institute for Food Inspection, Changde 415000, China
4 Laboratory of Stem Cell Regulation with Chinese Medicine and Its Application, Hunan University of Chinese Medicine, Changsha 410208, China
* Correspondence: lqin1011@126.com (L.Q.); 003568@hnucm.edu.cn (X.Z.); Tel.: +86-731-88458238 (L.Q.); +86-731-88458234 (X.Z.); Fax: +86-731-88458227 (L.Q. & X.Z.)
† These authors contributed equally to this work.

List of Supplementary Materials

List of contents **Page**

Figure S1. The ^{1}H NMR spectrum of compound (**1**) in CD_3OD……….………………………………S3
Figure S2. The ^{13}C NMR spectrum of compound (**1**) in CD_3OD….…….….………….……………….……S3
Figure S3. The DEPT spectrum of compound (**1**) in CD_3OD….…………….………….…………….……S4
Figure S4. The ^{1}H NMR spectrum of compound (**2**) in CD_3OD……...……………………………S5
Figure S5. The ^{13}C NMR spectrum of compound (**2**) in CD_3OD…….……………………..S5
Figure S6. The DEPT spectrum of compound (**2**) in CD_3OD……………………………S6
Figure S7. The ^{1}H NMR spectrum of compound (**3**) in CD_3OD……….………………………………S7
Figure S8. The ^{13}C NMR spectrum of compound (**3**) in CD_3OD….…….….………….………….……S7
Figure S9. The DEPT spectrum of compound (**3**) in CD_3OD….…………….………….…………….……S8
Figure S10. The ^{1}H NMR spectrum of compound (**4**) in CD_3OD……….………………………………S9
Figure S11. The ^{13}C NMR spectrum of compound (**4**) in CD_3OD….…….….………….………….……S9
Figure S12. The DEPT spectrum of compound (**4**) in CD_3OD….…….….………….………….……S10
Figure S13. The ^{1}H NMR spectrum of compound (**5**) in CD_3OD……….………………………………S11
Figure S14. The ^{13}C NMR spectrum of compound (**5**) in CD_3OD….…….….………….………….……S11
Figure S15. The DEPT spectrum of compound (**5**) in CD_3OD….…….….………….………….……S12

Figure S1. The ^{1}H NMR spectrum of compound (**1**) in CD_3OD

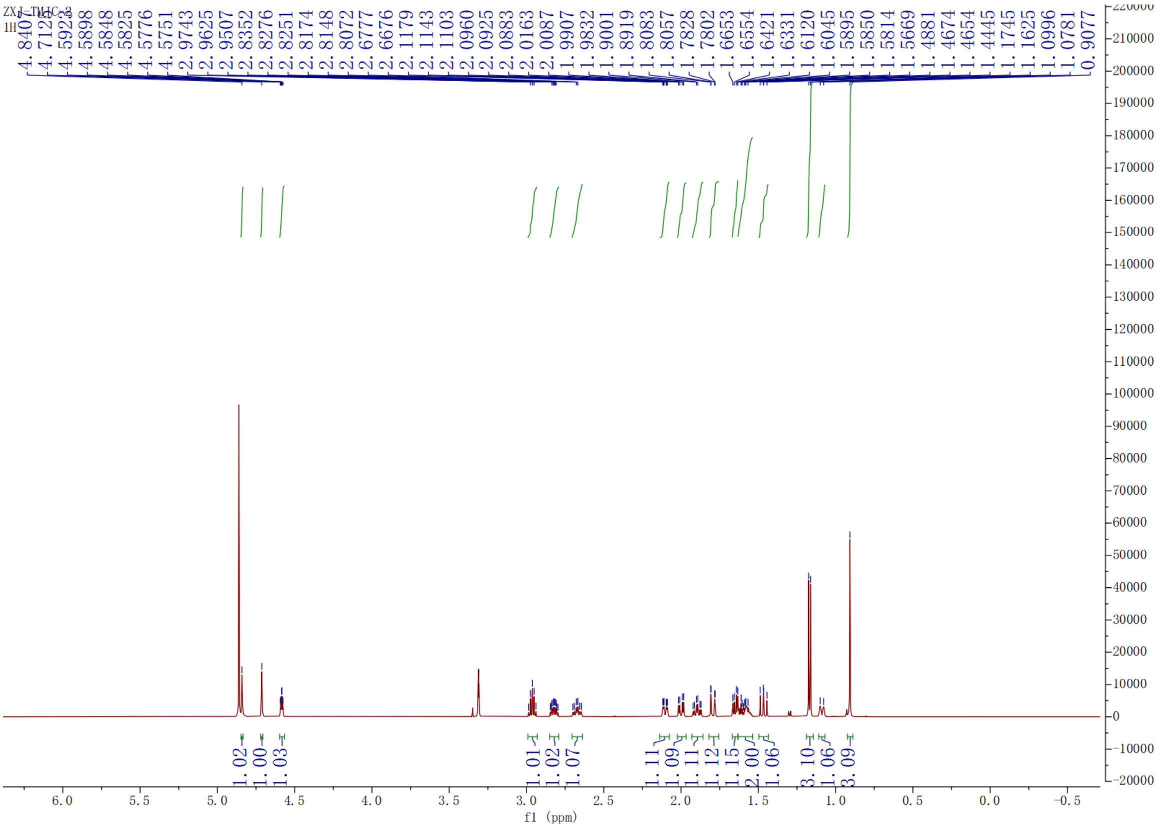

Figure S2. The ^{13}C NMR spectrum of compound (**1**) in CD_3OD

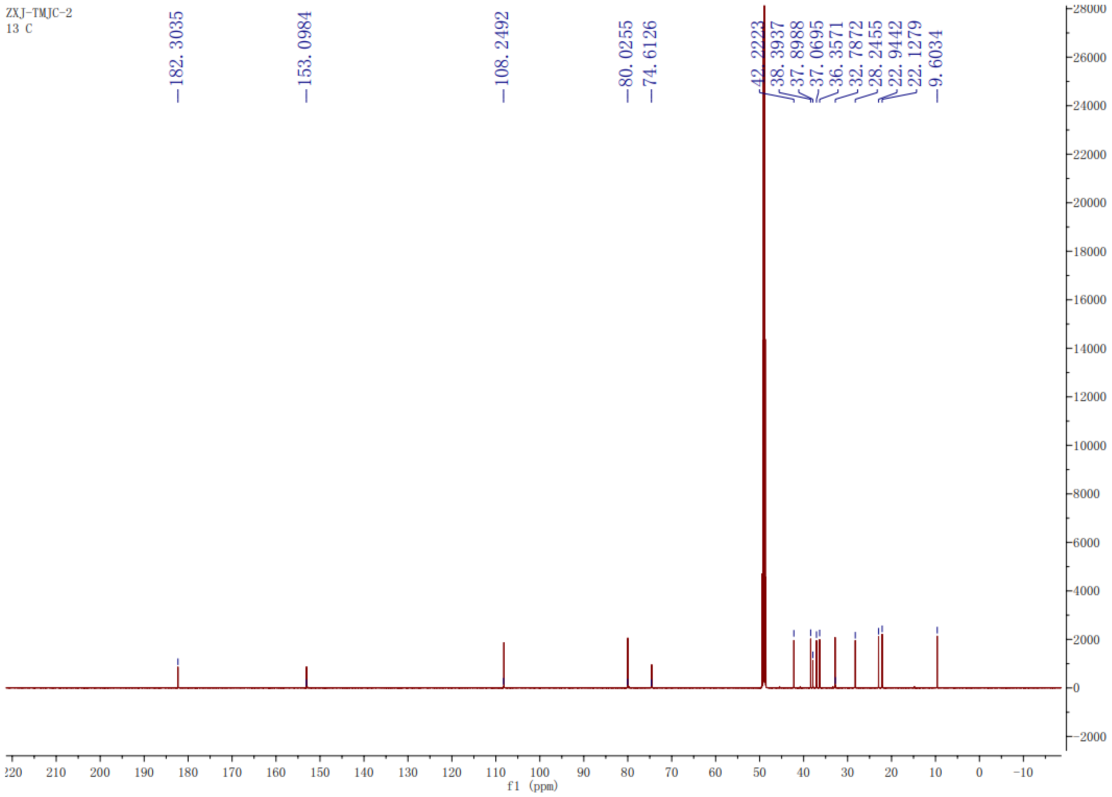

Figure S3. The DEPT spectrum of compound (**1**) in CD_3OD

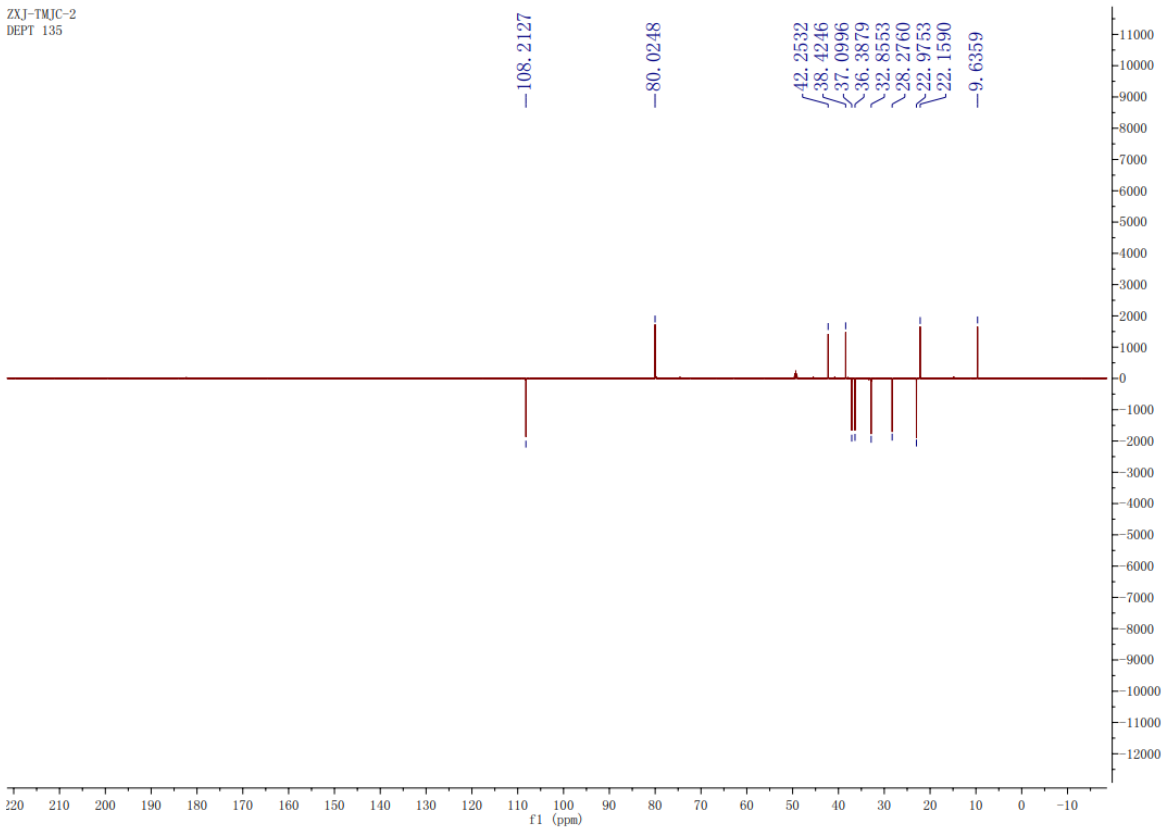

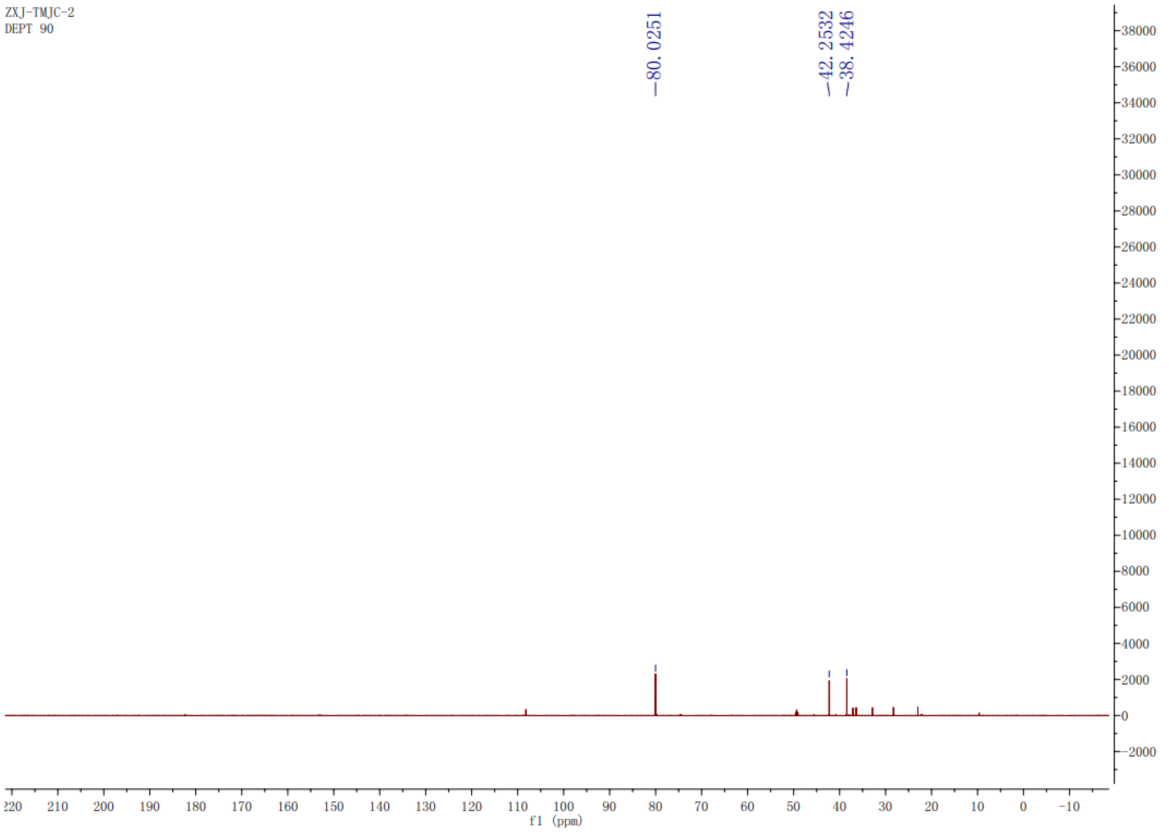

Figure S4. The ^{1}H NMR spectrum of compound (**2**) in CD_3OD

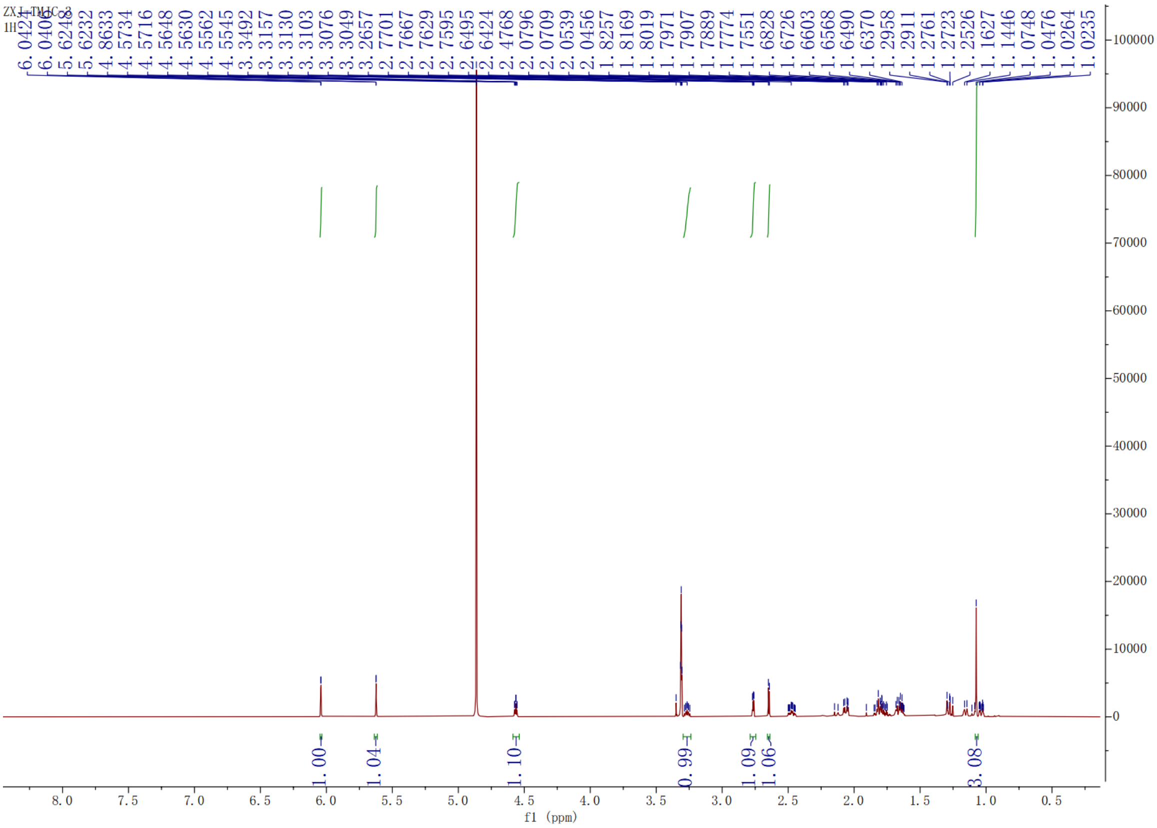

Figure S5. The ^{13}C NMR spectrum of compound (**2**) in CD_3OD

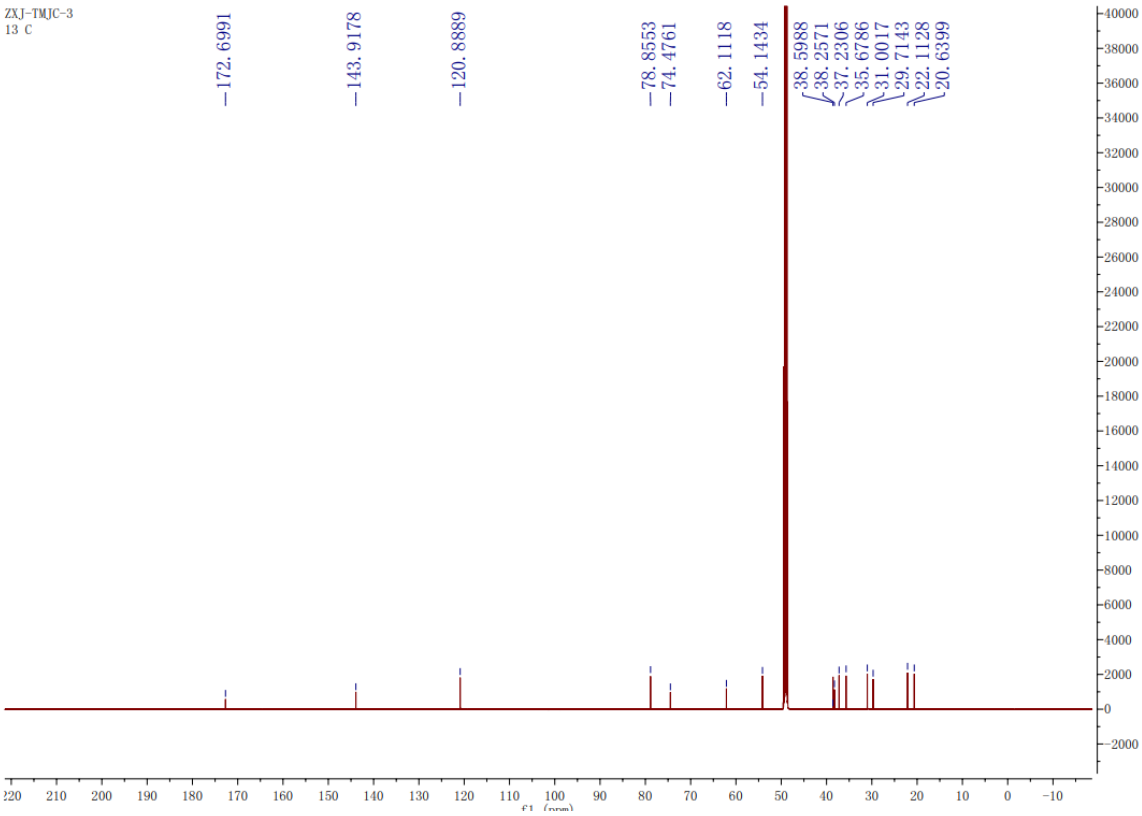

Figure S6. The DEPT spectrum of compound (**2**) in CD_3OD

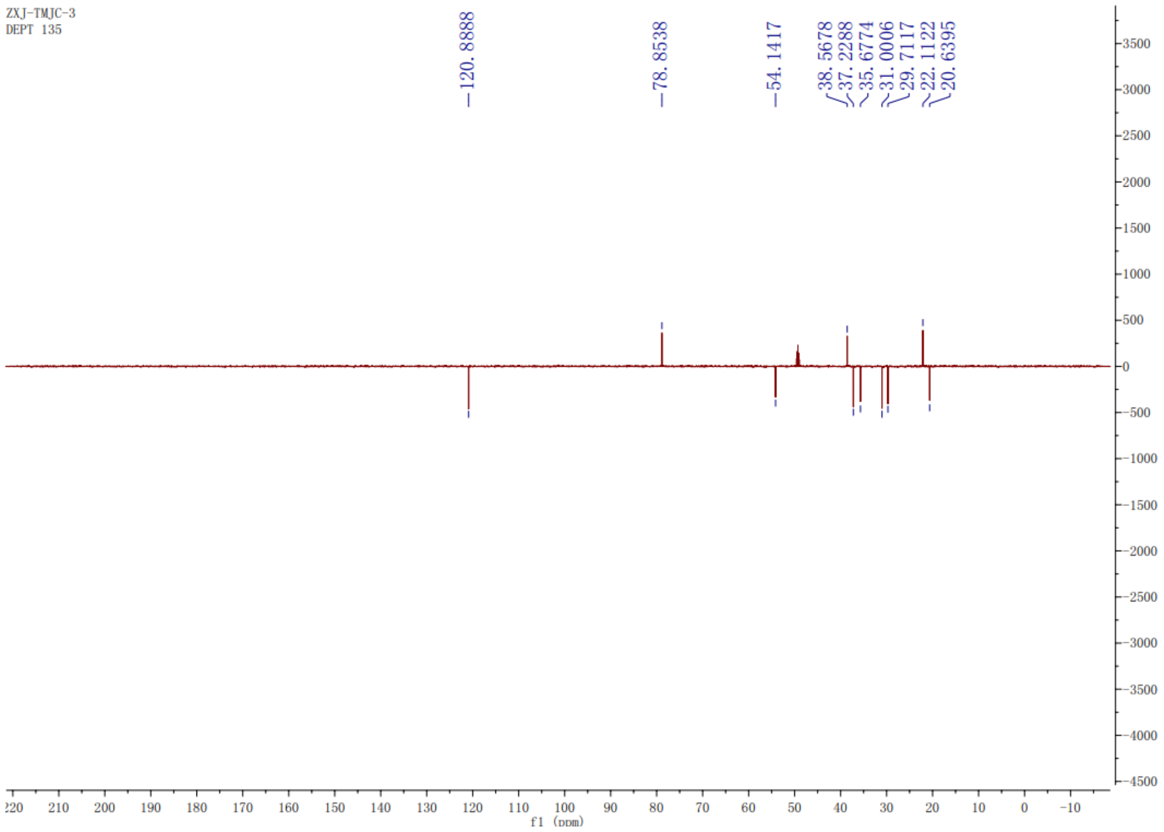

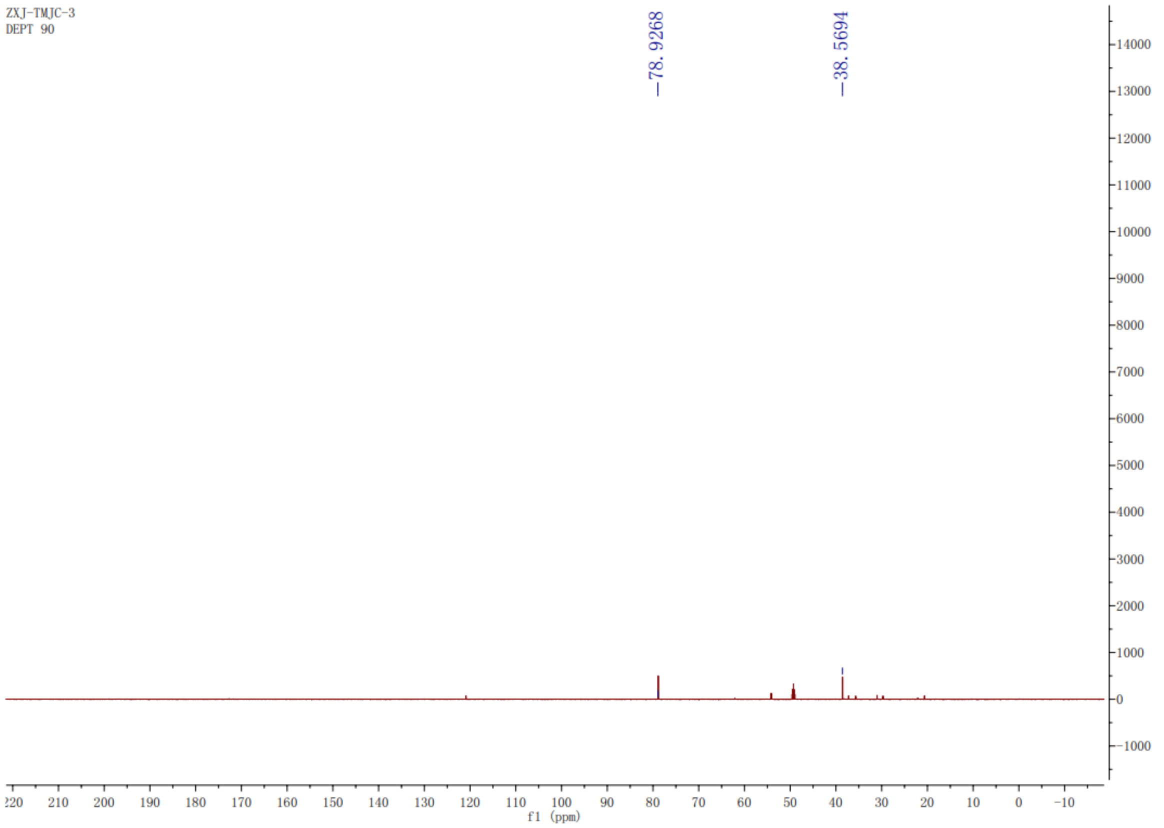

Figure S7. The ^{1}H NMR spectrum of compound (3) in CD_3OD

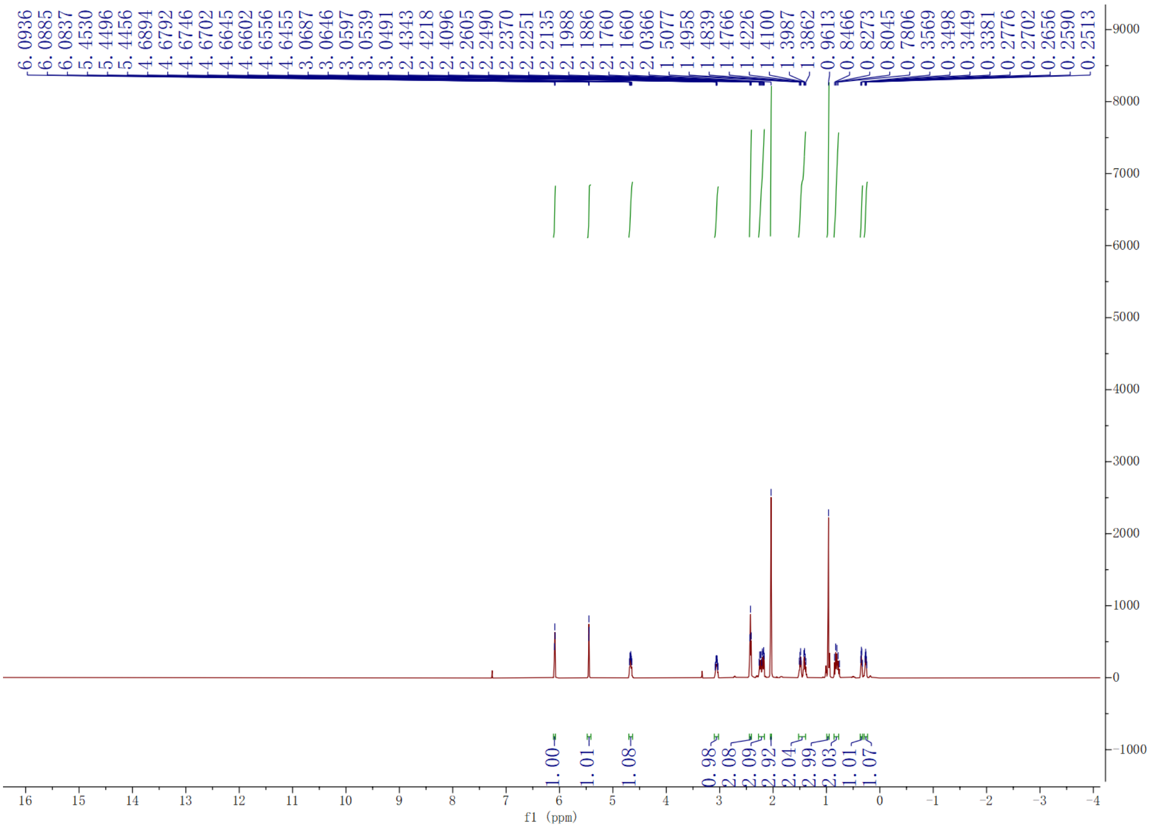

Figure S8. The ^{13}C NMR spectrum of compound (**3**) in CD_3OD

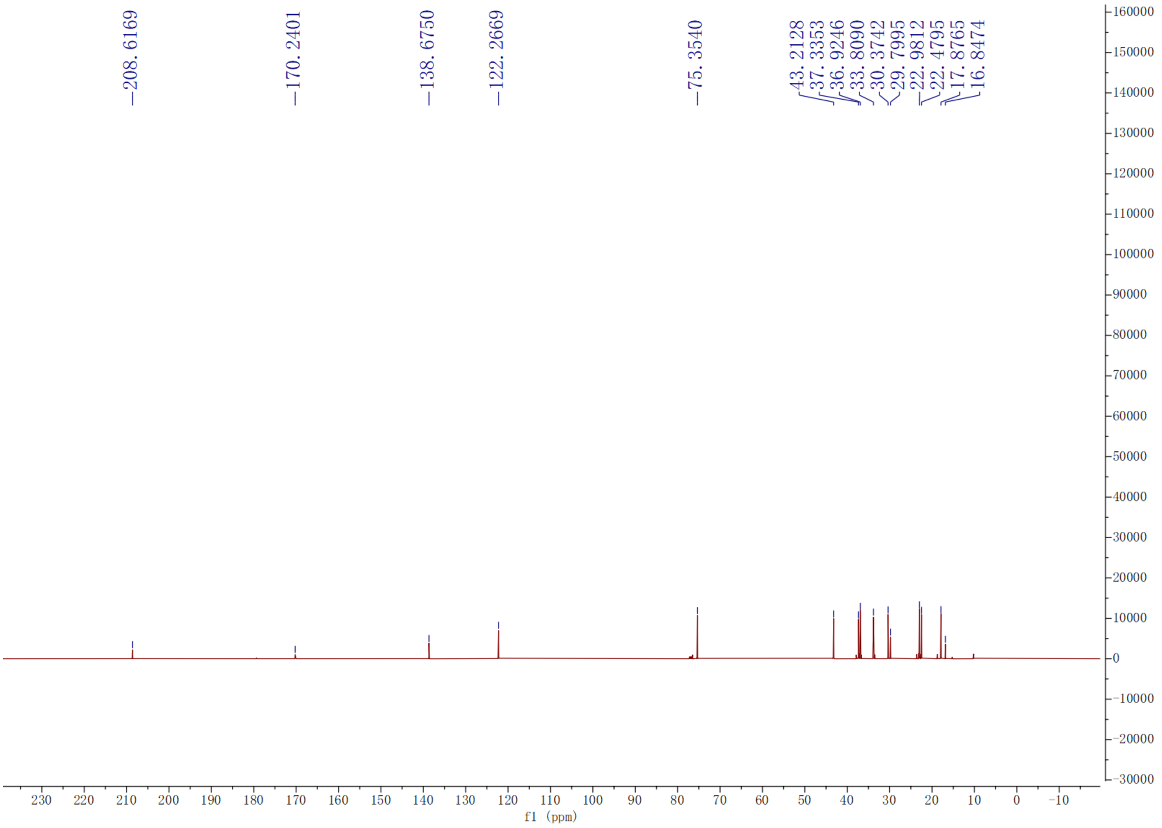

Figure S9. The DEPT spectrum of compound (**3**) in CD_3OD

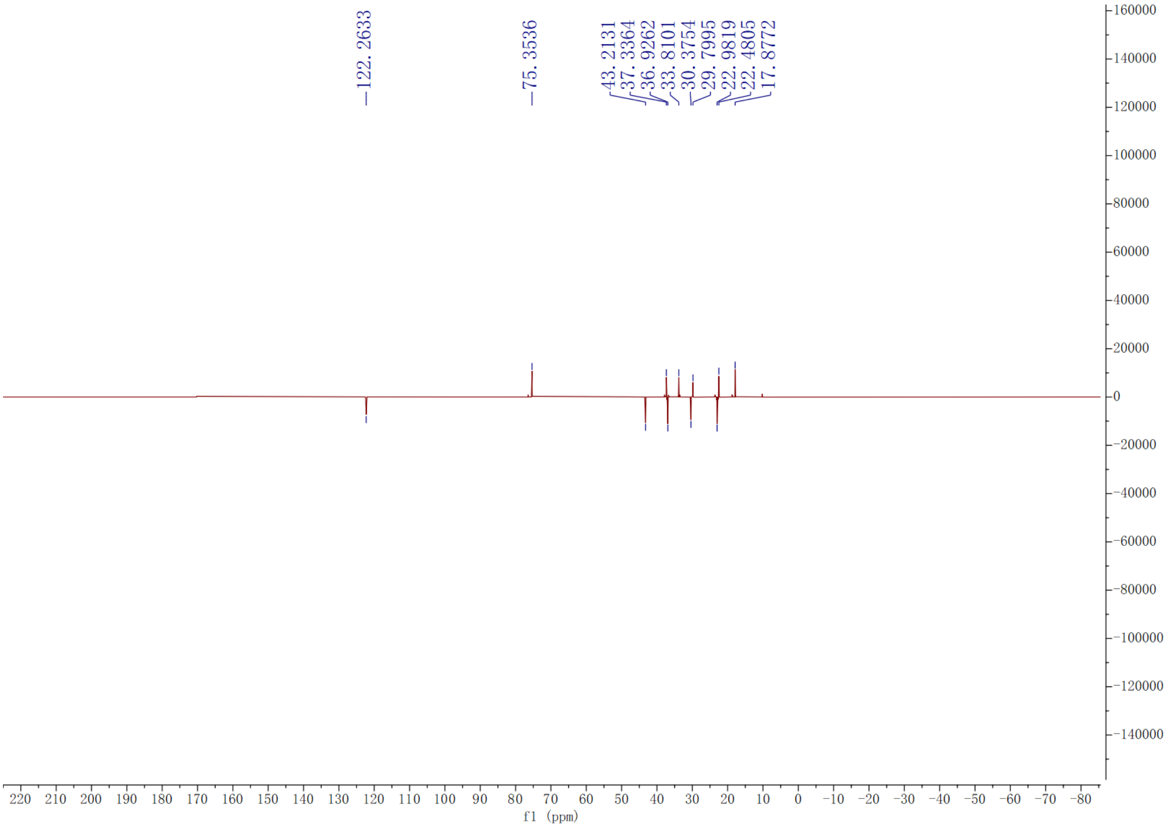

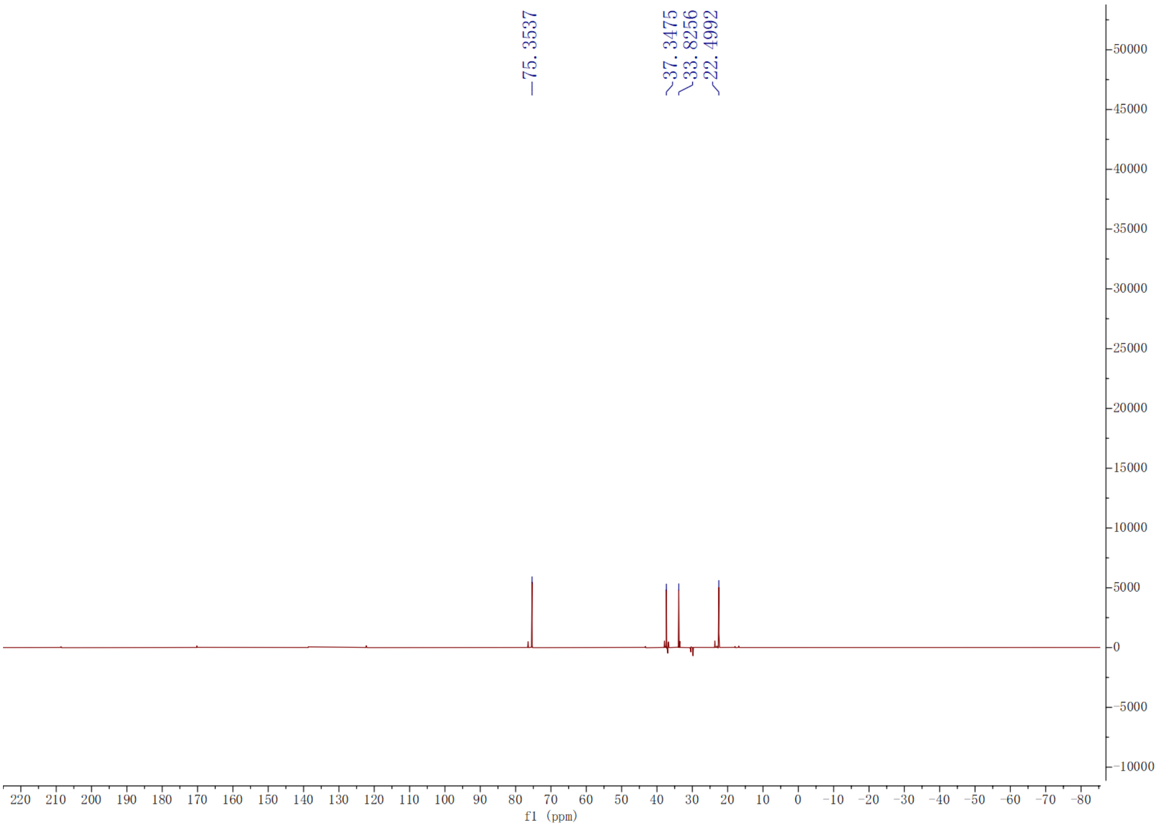

Figure S10. The ^{1}H NMR spectrum of compound (**4**) in CD_3OD

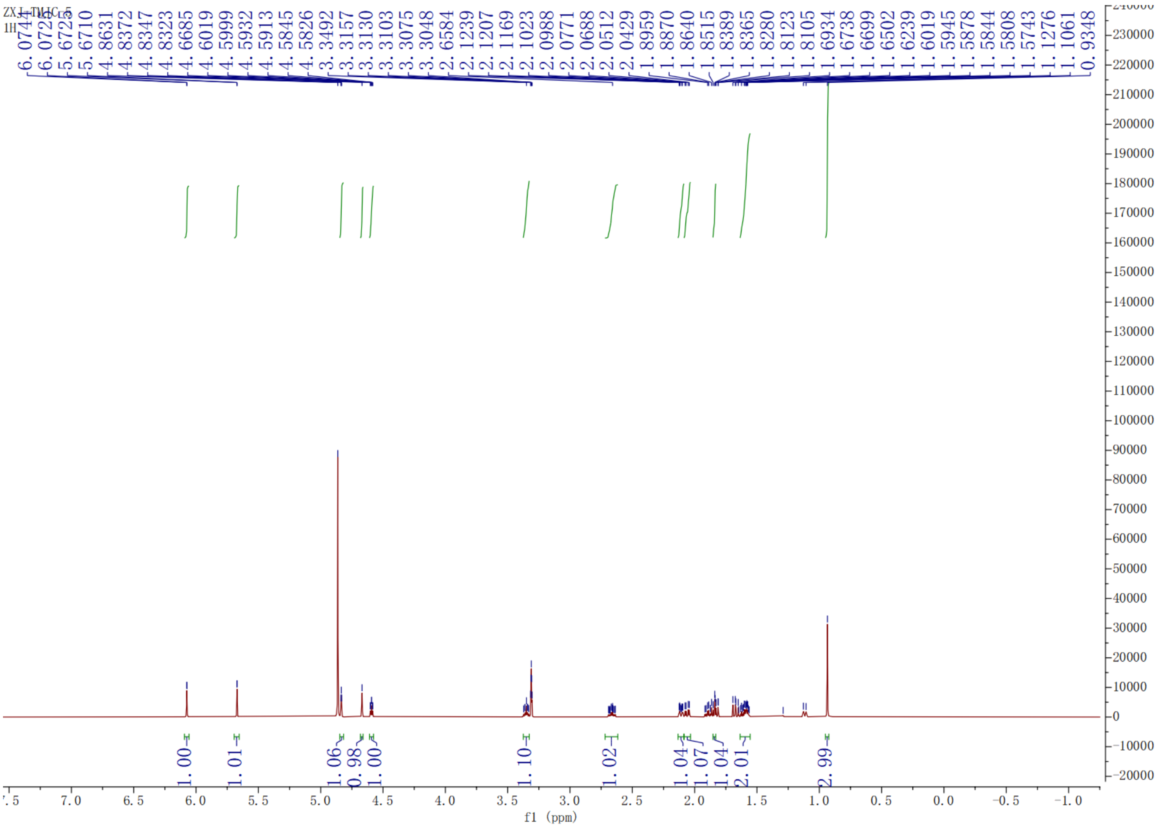

Figure S11. The ^{13}C NMR spectrum of compound (**4**) in CD_3OD

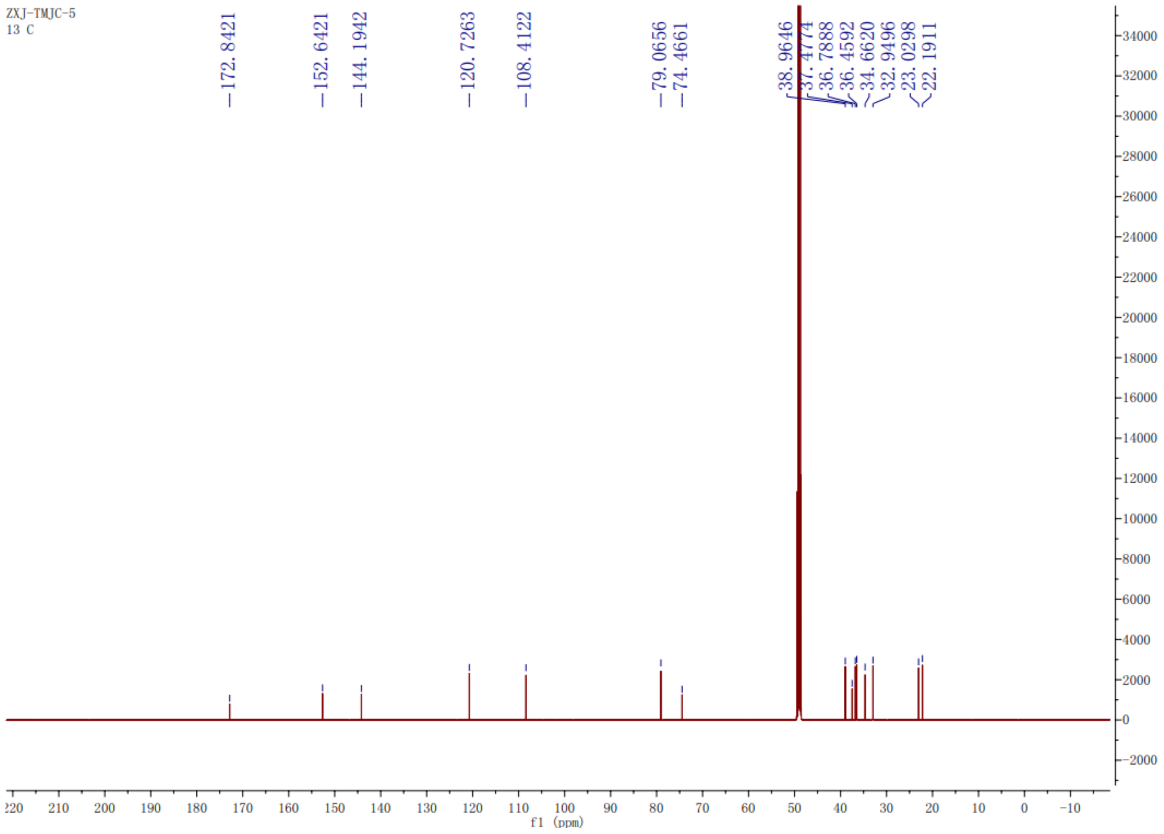

Figure S12. The DEPT spectrum of compound (**4**) in CD_3OD

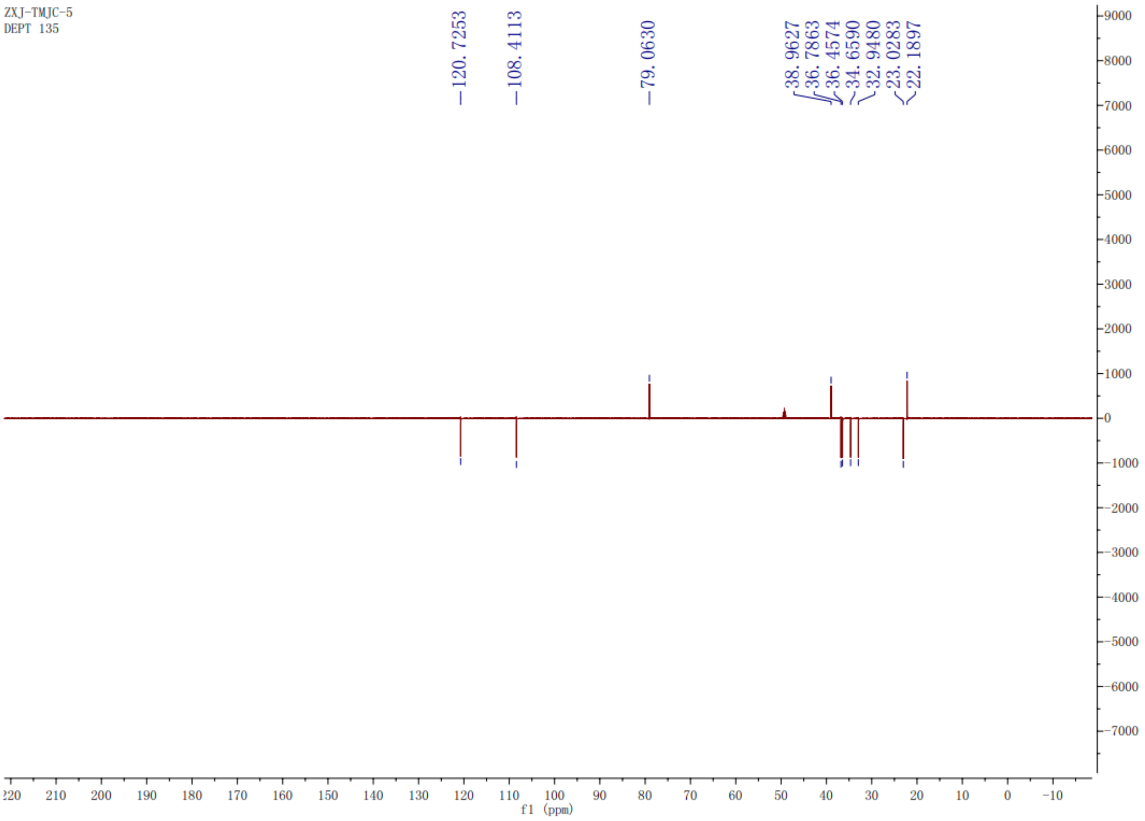

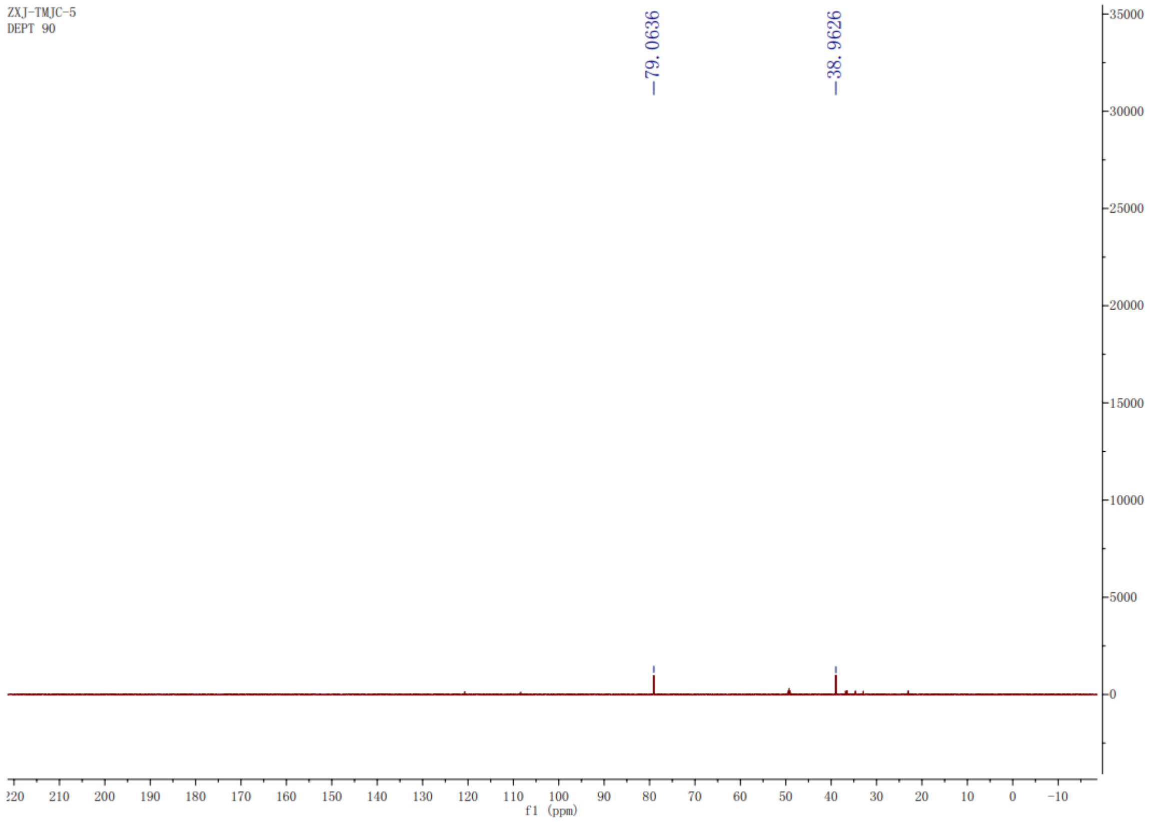

Figure S13. The ^{1}H NMR spectrum of compound (**5**) in CD_3OD

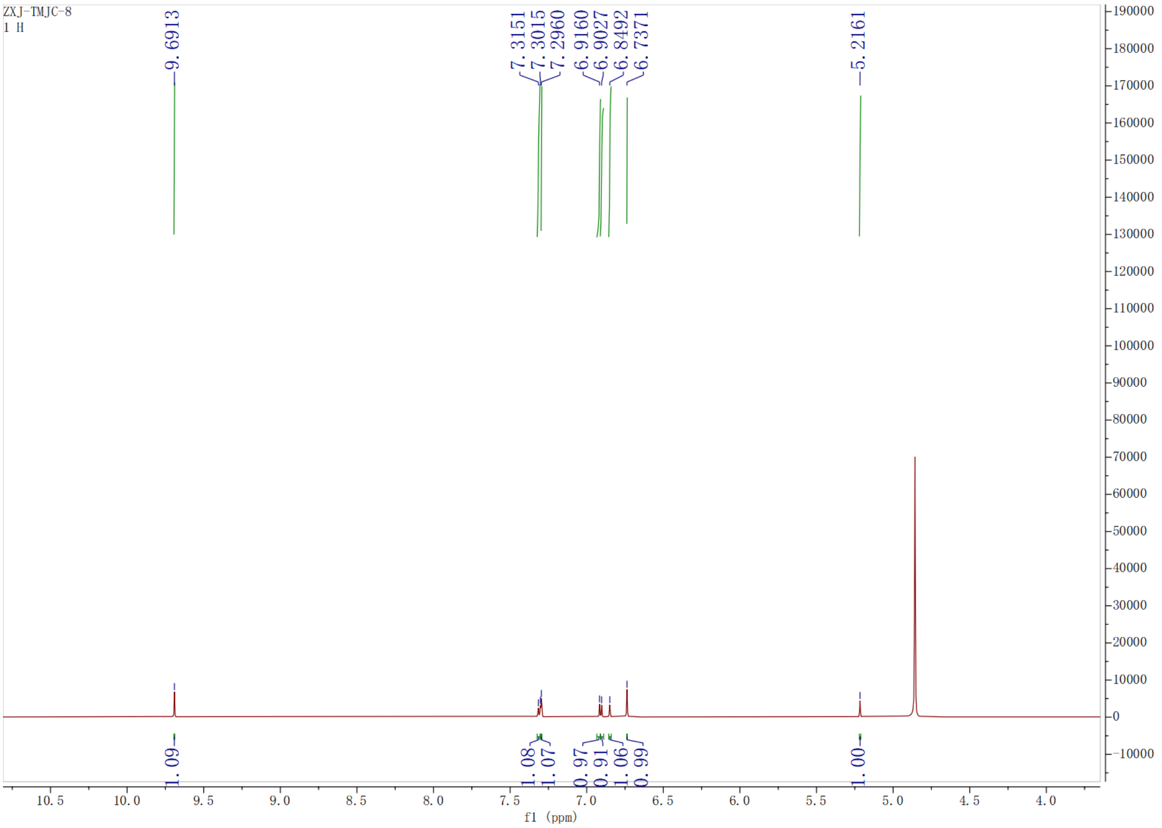

Figure S14. The ^{13}C NMR spectrum of compound (**5**) in CD_3OD

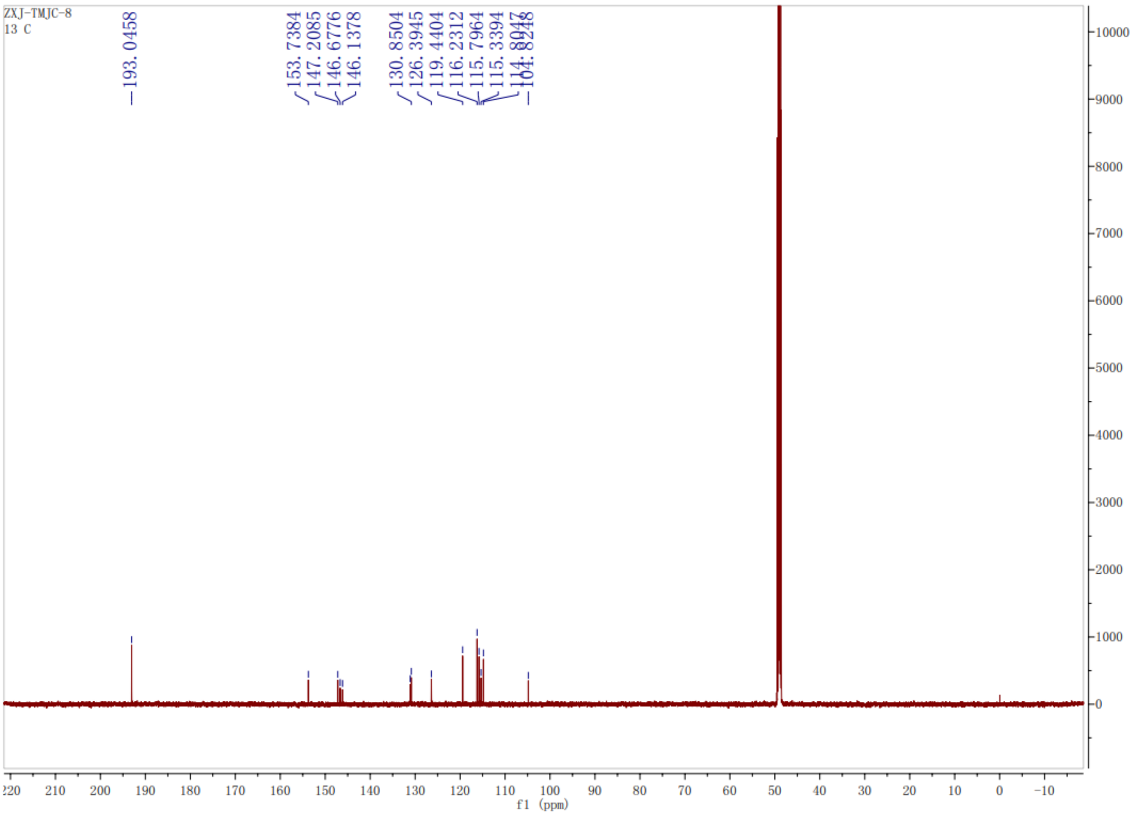

Figure S15. The DEPT spectrum of compound (**5**) in CD_3OD

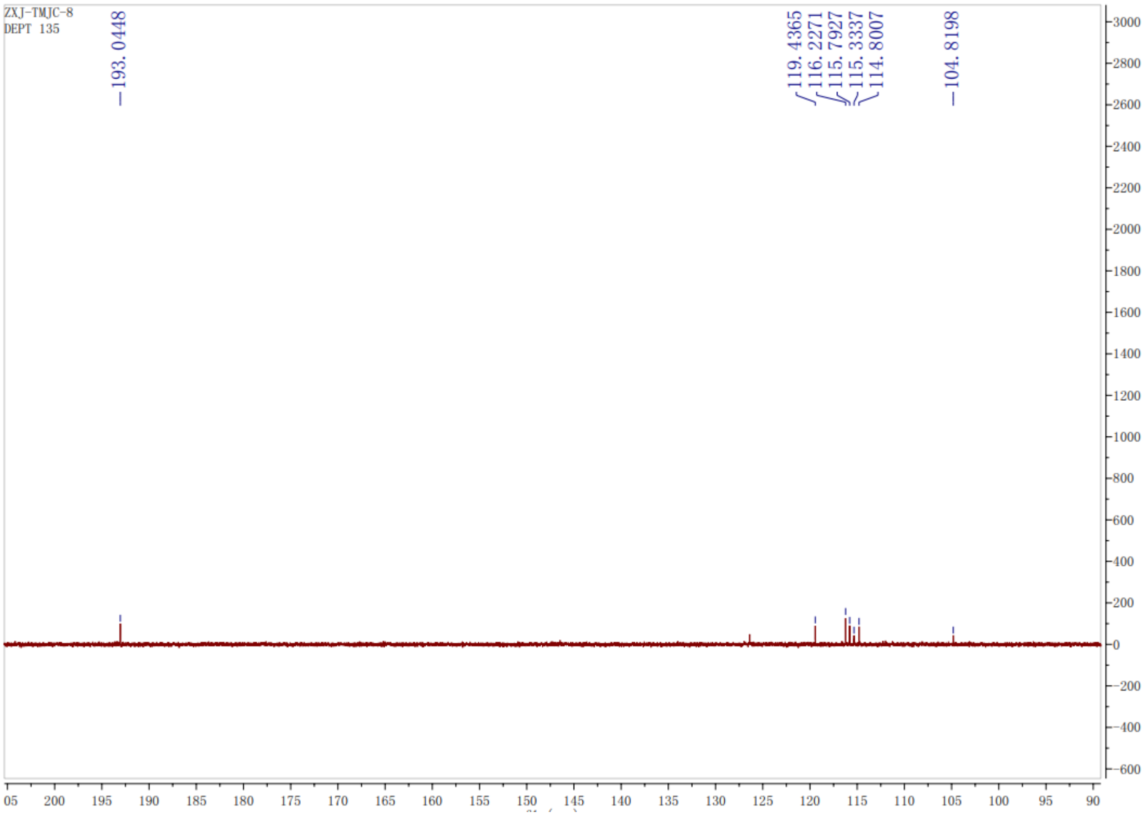

Supplement: Supplementary file 1 [file molecules-27-08313-s001.zip › molecules-2028474-supplementary.pdf]
